# Identification of De Novo *ZBTB18* Variant in a Patient With Global Developmental Delay, Seizures, and Juvenile Facies

**DOI:** 10.1155/ijpe/7226289

**Published:** 2026-05-23

**Authors:** Yan Wu, Bei Li, Liang Liu, Yan Wang, Dongjing Li

**Affiliations:** ^1^ Department of Neurology, Xi′an Children′s Hospital, Xi′an, Shaanxi, China

**Keywords:** developmental delay, intellectual developmental disorder autosomal dominant 22, whole exome sequencing, *ZBTB18*

## Abstract

**Background:**

*ZBTB18* encodes a C2H2‐type zinc finger protein that acts as a transcriptional repressor and is essential for neurodevelopment. Variants in *ZBTB18* are associated with intellectual developmental disorder, autosomal dominant 22 (MRD22). Patients with MRD22 present with intellectual disability (ID), corpus callosum anomalies, hypotonia, microcephaly, growth problems, epilepsy, and variable facial dysmorphism.

**Methods:**

Whole exome sequencing (WES) was performed to identify the molecular etiology. Sanger sequencing was used to validate the variant. Clinical assessments were conducted using brain magnetic resonance imaging (MRI) and electroencephalogram (EEG).

**Results:**

Our patient presented with sleep‐associated focal motor seizures, characterized by right‐sided clonic movements with impaired consciousness, accompanied by atypical absence seizures. She also exhibited global developmental delay, unexplained ankle hypertonia, and unique juvenile facies (characterized by a lack of complex emotional expression, a fixed gaze, and an overall immature facial gestalt). Video EEG demonstrated hypsarrhythmia, whereas brain MRI revealed no structural abnormalities at the age of 2.5 years. The patient received antiepileptic therapy, and seizure control was successfully achieved following perampanel treatment. A de novo *ZBTB18* variant (NM_205768.3: c.1474delA (p.Arg492Aspfs∗11)) was identified in our patient.

**Conclusion:**

To conclude, we report a de novo *ZBTB18* frameshift variant (NM_205768.3: c.1474delA (p.Arg492Aspfs∗11)) in a female patient presenting with global developmental delay, epilepsy, ankle hypertonia, and unique juvenile facies. Notably, seizures were ultimately controlled with perampanel, which may provide a therapeutic insight for drug‐resistant epilepsy in MRD22. Our study expands the phenotypic and genetic spectrum of *ZBTB18* and provides novel insights into the clinical management of MRD22.

## 1. Introduction

Intellectual developmental disorder, autosomal dominant 22 (MRD22, OMIM: 612337) is a neurodevelopmental disorder characterized by intellectual disability (ID), corpus callosum anomalies, hypotonia, microcephaly, growth problems, epilepsy, and variable facial dysmorphism [1]. MRD22 is caused by heterozygous mutations in the *ZBTB18* gene (NM_205768.3, OMIM: 608433) [2].

*ZBTB18*, located on chromosome 1q44, encodes a C2H2‐type zinc finger protein that functions as a transcriptional repressor. The 531‐amino acid protein comprises an N‐terminal POZ domain, a unique central region, and a C‐terminal domain containing four C2H2‐type zinc finger domains [3]. *ZBTB18* is expressed across various tissues, including the brain, skeletal muscle, pancreas, testis, and spleen. It was first identified to play an important role in cortical and cerebellar development [4, 5]. In mice, *ZBTB18* is essential for neurodevelopment. *ZBTB18* controls cell division of progenitor cells, neuronal differentiation, and regulates the survival of postmitotic cortical neurons [6, 7]. Furthermore, *ZBTB18* also directly modulates proneurogenic gene expression during neuronal differentiation and brain expansion [8]. Loss of *ZBTB18* leads to defective neuronal migration within the embryonic cerebral cortex [9], whereas reduction of *ZBTB18* expression in humans has been associated with cognitive impairment and tumorigenesis in the brain [5]. To date, 33 pathogenic *ZBTB18* variants have been reported, including 8 nonsense, 13 missense, and 12 frameshift variants.

Here, we report a 6‐year‐old female patient who presented with global developmental delay, seizures, ankle hypertonia, and juvenile facies. Seizure control was successfully achieved following perampanel treatment. Whole exome sequencing (WES) identified a novel de novo frameshift variant in the *ZBTB18* gene (NM_205768.3: c.1474delA (p.Arg492Aspfs∗11)). Our study expands the genotypic and phenotypic spectrums of *ZBTB18*‐associated MRD22 and provides a therapeutic insight for managing drug‐resistant epilepsy in MRD22.

## 2. Materials and Methods

### 2.1. Patient

Our patient underwent a series of clinical assessments, including brain magnetic resonance imaging (MRI) and electroencephalogram (EEG).

### 2.2. WES

The reference genomic DNA sequences used for the *Homo sapiens ZBTB18* gene were obtained from the NCBI GenBank database under Accession Number NM_205768.3. Genomic DNA was extracted from the peripheral blood of our patient and her parents. A DNA library was constructed and captured using the IDT xGen Exome Research Panel. Quality control was performed on the raw sequencing data (> 10 GB), requiring an average depth of > 100X and 20X coverage > 95%. Subsequently, clean reads were aligned to the human reference genome GRCh38/hg38 using BWA, Samtools, and Picard. Following alignment, variants were annotated using ANNOVAR. The public databases, such as ExAC, 1000Genomes, and gnomAD, were used to analyze population variation frequency. Multiple databases, such as dbSNP, OMIM, HGMD, and ClinVar datasets, were used to evaluate the variant pathogenicity. The protein function was predicted by SIFT, Polyphen2, and MutationTaster. Finally, variants associated with clinical phenotypes were categorized according to the ACMG guidelines [10]. Candidate variants identified by WES were confirmed using Sanger sequencing.

## 3. Results

### 3.1. Case Reports

The patient was the first child and was delivered following a full‐term cesarean section due to oligohydramnios. Her birth weight was 3.3 kg, and there was no documented fetal distress or birth asphyxia. Developmental milestones included independent sitting at 7 months, crawling at 12 months, and independent ambulation with the utterance of “papa” and “mama” at 18 months. However, expressive language was significantly delayed, with a limited vocabulary of only a few words at the age of 3 years and 10 months. The family history was unremarkable for similar conditions.

**Figure 1:**
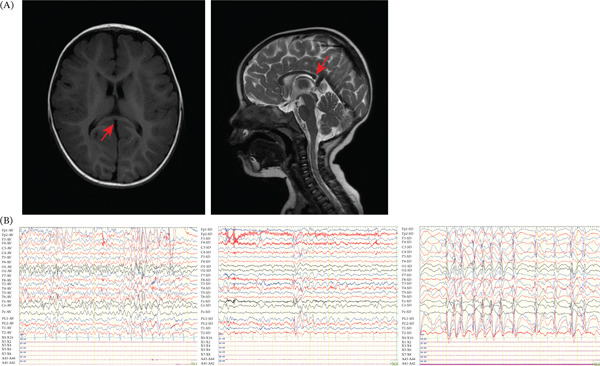
Brain MRI and EEG in our patient. (A) Brain magnetic resonance imaging in the patient. Brain MRI demonstrated thinning of the splenium of the corpus callosum at the age of 1 year (left), whereas follow‐up MRI showed a normal appearance of the splenium at the age of 2 years (right). (B) EEG in the patient. During the awake state, bilateral occipital regions show an 8–9‐Hz low‐to‐medium amplitude alpha rhythm, intermixed with a small amount of low‐amplitude fast activity. The rhythm is approximately symmetrical between hemispheres, with poor amplitude modulation (left); EEG during the waking period shows frequent generalized 1.5–2.5 Hz high to extremely high amplitude sharp and slow wave complexes, sometimes with left‐sided predominance; during the sleeping period, the EEG demonstrates generalized paroxysms of 1.5–2.5 Hz high to extremely high amplitude sharp and slow wave complexes. Focal epileptiform discharges with left‐sided predominance.

At 1 year of age, the patient was diagnosed with global developmental delay and malnutrition at the department of rehabilitation in our hospital. At that time, brain MRI revealed thinning of the splenium of the corpus callosum (Figure 1A). At 2.5 years of age, she developed sleep‐associated focal motor seizures, characterized by right upper limb clonic movements, binocular strabismus, and impaired consciousness. These episodes typically lasted approximately 20 s and progressed to twitching of the right upper and lower limbs accompanied by drooling. Based on the clinical presentation and video EEG, she was diagnosed with focal impaired awareness motor‐onset seizure and global developmental delay. Subsequent brain MRI (plain scan and ASL) revealed no significant structural abnormalities. The EEGs demonstrated frequent epileptiform discharges and slow wave activity in the left middle temporal and parietal regions during wakefulness.

At 2 years and 10 months of age, the patient developed recurrent nocturnal motor seizures. Initial treatment with lacosamide (7.5 mg/kg/day) and valproic acid (18 mg/kg/day) failed to achieve seizure control. A brief trial of perampanel (0.5 mg/day) was discontinued by the parents after 2 weeks due to an increase in seizure frequency. Video EEG revealed multifocal and widespread sharp waves, sharp slow wave complexes, and slow wave activity, predominantly originating from the left hemisphere (Figure 1B). Subsequently, the addition of zonisamide (up to 11 mg/kg/day) also proved to be ineffective. Given the high NREM discharge index (80%) and the co‐occurrence of focal and absence seizures, perampanel was reintroduced [11, 12]. The dosage of perampanel was gradually titrated from 0.5 mg/day to 2 mg/night. Following the discontinuation of zonisamide and lacosamide, sustained seizure control was successfully achieved (Table S1).

Our patient is 6 years old currently. She can navigate stairs, albeit with an awkward gait. Although she is capable of forming sentences, she exhibits significant impairment in logical reasoning and disorganized thinking. She presents with juvenile facies (characterized by a lack of complex emotional expression, a fixed gaze, and an overall immature facial gestalt), microcephaly, and hypertonia in the ankles, with Level 4 muscle strength on physical examination. Cognitive function was assessed using the Wechsler Intelligence Scale for Children (WISC). The results showed a verbal IQ of 53 (score: 14), a performance IQ of 56 (score: 21), and a full‐scale IQ of 49 (total score: 35).

### 3.2. Identification of *ZBTB18* Variant

**Figure 2:**
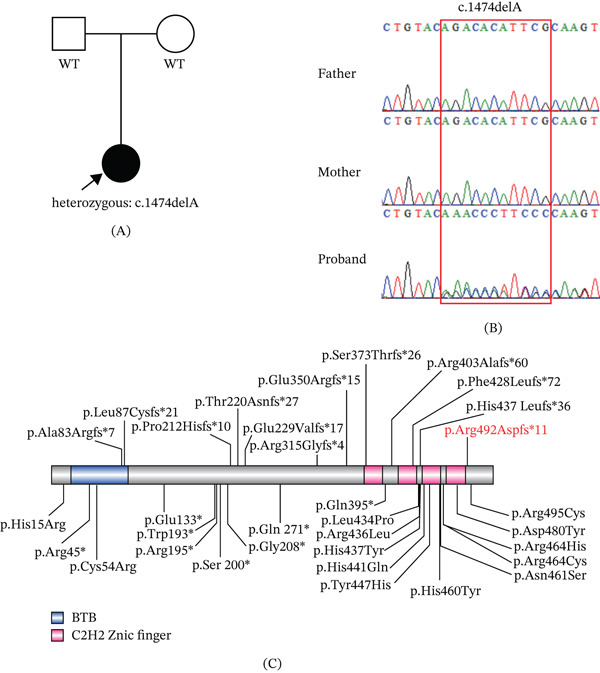
The variant in the ZBTB18 gene was successfully identified. (A) The pedigree of the patient under examination. The proband is indicated by a filled shape with an arrow. (B) Sanger sequencing of the family. The de novo variant was identified through sequencing. (C) Schematic of protein structure and reported variants in the ZBTB18 gene. The variant identified in our case is highlighted in red text.

WES was performed to elucidate the genetic etiology in this case. A novel heterozygous *ZBTB18* variant (NM_205768.3: c.1474delA (p.Arg492Aspfs∗11)) was identified, and Sanger sequencing confirmed that the variant was de novo (Figure 2A,B). This variant is rare and is not included in public population databases (gnomAD and ExAC) and Clinvar. The c.1474delA variant induces a frameshift starting at Codon 492, substituting arginine with asparagine and creating a premature stop Codon 11 amino acids downstream. This is predicted to result in a truncated protein. In accordance with the ACMG guidelines, this variant was classified as “pathogenic” (PVS1_STRONG+PS2+PM2_STRONG). The spectrum of *ZBTB18* gene variants was summarized in Figure 2C, and the clinical features of previously reported cases were reviewed in Table 1.

**Table 1:** Genotype–phenotype characteristics of patients with the *ZBTB18* variant.

References	Gender (male/female) and onset age	*ZBTB18* mutation	Inheritance	Intellectual disability	Corpus callosum anomalies	Seizure	Seizure type	Antiseizure medications tried	Microcephaly	Hypotonia	Variable facial dysmorphism	Others
Our patient	F, 2.5 years	c.1474delA (p.Arg492Aspfs∗11)	De novo	+	—	+	Focal motor seizures/atypical absence seizures	Perampanel	+	Ankle hypertonia	—	Juvenile facies
Rauch A. et al. [13]	F, 18 years	c.1483C>G (p.Arg495Cys)	De novo	+	—	NA	NA	NA	—	NA	NA	NA
de Munnik et al. [14]	F, 2.8 years	c.397G>T (p.Glu133∗)	De novo	+	—	—	—	—	+	—	+	Gastrointestinal problems
Lopes et al. [15]	F, 5 years	c.583C>T (p.Arg195∗)	De novo	+	—	NA	NA	NA	+	+	—	Bruxism
McRae et al. [16]	M, NA	c.622G>T (p.Gly208∗)	De novo	+	NA	NA	NA	NA	2/5 with microcephaly	NA	2/5 with abnormality of nasal alae	2/5 with clinodactyly of fifth finger, 3/5 with abnormality of hair or hair pattern
	F, NA	c.635del (p.Pro212Hisfs∗10)	De novo									
	M, NA	c.811C>T (p.Gln271∗)	De novo									
	M, NA	c.1046dup (p.Glu350Argfs∗15)	De novo									
	F, NA	c.1391G>A (p.Arg464His)	De novo									
Cohen JS. et al. [2]	M, 3 years	c.943_944del (p.Arg315Glyfs∗4)	De novo	+	+	—	—	—	—	+	+	Strabismus
	M, 7 years	c.1382A>G (p.Asn461Ser)	NA	+	+	—	—	—	—	+	—	
	M, 4 years	c.1183C>T (p.Gln395∗)	De novo	+	+	—	—	—	+	+	+	Strabismus, single febrile seizure
	M, 34 years	c.1390C>T (p.Arg464Cys)	De novo	+	NA	—	—	—	—	—	+	Drooling
	M, 6 years	c.133C>T (p.Arg45∗)	De novo	+	+	+	Focal motor seizures without impairment of consciousness as well as focal seizures with impairment of consciousness.	Levetiracetam	—	+	+	—
	M, 15 years	c.1118del (p.Ser373Thrfs∗26)	NA	+	NA	NA	NA	NA	—	NA	—	—
	NA	c.160T>C (p.Cys54Arg)	De novo	+	—	—	—	—	+	—	+	—
Depienne C. et al. [17]	M, 14 years	c.44A>G (p.His15Arg)	De novo	+	+	—	—	—	—	+	NA	—
	F, 12 years	c.599del (p.Ser200∗)	De novo	+	+	+	Tonic–clonic seizures and head turning with cyanosis	NA	—	—	NA	—
	M, 23 years	c.1301T>C (p.Leu434Pro)	De novo	+	NA	+	Tonic–clonic seizures and head turning with cyanosis	NA	—	—	NA	—
Van der Schoot V. et al. [18]	M, 15 years	c.1339T>C (p.Tyr447His)	De novo	+	—	+	NA	NA	—	+	+	—
	M, 1 year	c.579G>A (p.Trp193∗)	De novo	+	+	—	—	—	+	—	+	—
	M, 13 years	c.259del (p.Leu87Cysfs∗21)	De novo	+	—	—	—	—	—	—	+	—
Wang et al. [19]	M, 12 years	c.658dup (p.Thr220Asnfs∗27)	De novo	NA	NA	NA	NA	NA	NA	NA	NA	—
	M, 7 years	c.686_687del (p.Glu229Valfs∗17)	De novo	NA	NA	NA	NA	NA	NA	NA	NA	—
	M, 17 years	c.246dup (p.Ala83Argfs∗7)	De novo	NA	NA	NA	NA	NA	NA	NA	NA	—
	M, 18 years	c.1279_1307dup (p.His437Leufs∗36)	Unknown	NA	NA	NA	NA	NA	NA	NA	NA	—
	F, 6 years	c.1307G>T (p.Arg436Leu)	De novo	+	NA	NA	NA	NA	NA	NA	+	—
	F, 1 year	c.1378C>T (p.His460Tyr)	De novo	+	NA	+	Infantile spasms	NA	NA	NA	NA	Visual impairment, hypsarrhythmia
	F, 3.7 years	c.1438G>T (p.Asp480Tyr)	De novo	+	+	NA	NA	NA	NA	+	NA	Brother with developmental delay and hypotonia.
Li et al. [20]	NA	c.1323C>G (p.His441Gln)	De novo	+	NA	NA	NA	NA	NA	NA	NA	—
Zhang et al. [21]	M, 3.7 years	c.1282_1283del (p.Phe428Leufs∗72)	De novo	+	—	+	Generalized tonic–clonic seizure	Levetiracetam	—	—	—	—
Pande S. et al. [22]	M, 5 years	c.1309C>T (p.his437Tyr)	De novo	NA	NA	NA	NA	NA	NA	NA	NA	NA
Yang et al. [23]	M, NA	c.1207delC (p.Arg403Alafs∗60)	De novo	+	—	—	—	—	—	+	+	Thyroid dysfunction, periventricular, white matter abnormalities

## 4. Discussion

*ZBTB18* (also known as ZNF238 or RP58), located on chromosome 1q44, encodes a zinc finger transcription factor that functions as a transcriptional repressor pivotal for cortical and cerebellar development [24, 25]. Xiang et al. found that conditional knockout of *ZBTB18* in mice resulted in microcephaly, reduced cortex thickness, agenesis of the corpus callosum, and cerebellar hypoplasia in the nervous system, resembling the clinical manifestations of 1q43–q44 deletion syndrome [8, 17]. Recent evidence further indicates that heterozygotic *ZBTB18* missense and truncating variants impair excitatory synaptic maturation, contributing to the cognitive dysfunction associated with *ZBTB18* haploinsufficiency [26].

In humans, pathogenic variants in *ZBTB18* disrupt neurodevelopment, contributing to MRD22 (OMIM: 612337), which is characterized by impaired intellectual development with frequent cooccurrence of corpus callosum anomalies, hypotonia, microcephaly, growth problems, and variable facial dysmorphism [18]. To date, including the present case, 33 *ZBTB18* variants have been reported (Table 1), comprising 13 missense, 8 nonsense, and 12 frameshift variants. Herein, we describe a novel de novo *ZBTB18* frameshift variant (NM_205768.3: c.1474delA (p.Arg492Aspfs∗11)) in a patient exhibiting global developmental delay, seizures, unexplained ankle hypertonia, and juvenile facies. This case provides novel insights that extend beyond the current phenotypic and therapeutic understanding of this condition.

Our patient manifested the classical features of MRD22, including global developmental delay, seizures, microcephaly, and corpus callosum hypoplasia, which is consistent with previous studies [14, 18, 21]. Corpus callosum hypoplasia has been widely recognized as the core neuroimaging feature of MRD22. Cohen et al. identified five patients with *ZBTB18* variants, four of whom presented with various degrees of corpus callosum hypoplasia, including one global corpus callosum hypoplasia, two posterior corpus callosum hypoplasia, and one mild global corpus callosum hypoplasia with possible dysplasia [2]. Additionally, Yang et al. noted that 58.8% of reported patients with *ZBTB18* variants exhibited corpus callosum hypoplasia [23]. In contrast, the dynamic nature of the corpus callosum morphology in our patient presents another intriguing finding. The thinning of the splenium observed at 1 year of age resolved on follow‐up MRI at 2.5 years of age. This dynamic change likely reflects an age‐dependent developmental lag or delayed myelination rather than a permanent structural defect. The morphological development of the corpus callosum is influenced by myelination. Myelination of the corpus callosum begins in the anterior regions during infancy and progresses posteriorly, with the splenium undergoing significant myelination during the second year of life and continuing into adolescence. Notably, brain MRI may show age‐related changes in callosal thickness and signal intensity that reflect ongoing myelination rather than structural abnormalities [27–29]. Furthermore, our report offers a detailed electroclinical characterization of *ZBTB18*‐related epilepsy and suggests a potential therapeutic strategy. To date, only two MRD22 patients have been reported to achieve seizure control with levetiracetam. One patient presented with focal seizures, whereas the other one displayed generalized tonic–clonic seizures. Distinct from these cases, our patient exhibited a combination of focal motor seizures and atypical absence seizures, and both seizures were controlled with perampanel. As a selective AMPA‐type glutamate receptor antagonist, perampanel holds promise as a treatment option for both focal motor seizures and atypical absence seizures [11, 30]. Its efficacy in our case suggests a potential role for aberrant glutamatergic signaling in the pathophysiology of seizures due to *ZBTB18* haploinsufficiency. Interestingly, Nagano et al. found significantly reduced expression of the GluA1 (an essential AMPA receptor subunit) in the cerebral cortex of ZBTB18^+/−^ mouse models, further supporting this hypothesis [26]. Although the precise link between *ZBTB18* and AMPA receptor function is unexplored, the established role in neuronal maturation and synaptic function provides a plausible connection. To our knowledge, this is the first detailed report of perampanel use in MRD22. These findings provide a compelling rationale for considering AMPA receptor antagonists in the management of drug‐resistant epilepsy in this population, warranting further clinical attention.

Interestingly, the patient experienced a transient increase in seizure frequency during the initial introduction of perampanel in combination with lacosamide for antiepileptic treatment. Several factors may account for this observation. First, the dosage factor must be considered. A retrospective study conducted in Nanjing Children′s Hospital indicated that the recommended starting dose of perampanel is 0.5 mg/day and the effective maintenance dose typically ranges from 2 to 8 mg/day for pediatric patients weighing < 20 kg or aged < 4 years [31]. However, in our patient′s first trial, the dosage was maintained at only 0.5 mg/day, which is significantly below the therapeutic maintenance level. This may potentially lead to failure to achieve adequate seizure antiepileptic efficacy. Second, clinical evidence suggests that perampanel monotherapy often yields superior efficacy compared with adjunctive therapy [31]. In the patient′s first trial, perampanel was administered in combination with lacosamide. This multidrug regimen, particularly in refractory seizures, might have limited the clinical effectiveness of perampanel. Third, drug‐resistant epilepsy is characterized by spontaneous fluctuations in seizure frequency, independent of medication changes [32]. Notably, the eventual achievement of seizure control upon reintroducing perampanel at 2 mg/day (monotherapy) underscores that achieving the recommended maintenance threshold is critical for therapeutic success in managing the specific seizure types observed in our patient.

Notably, the neurological examination revealed paradoxical ankle hypertonia, a feature that contrasts with the hypotonia commonly reported in MRD22 patients [2, 15, 17]. This finding highlights the significant phenotypic variability of motor system involvement in this disorder. Such hypertonia could suggest an upper motor neuron component or a dysfunction of inhibitory circuits within the corticospinal system. This specific aspect of motor dysfunction has not been thoroughly investigated in MRD22 and warrants further attention.

Additionally, although typical facial dysmorphism (e.g., round face, prominent forehead, and flat nasal bridge) is well‐documented in previous studies [14, 19], our patient did not manifest these classic features. Instead, she exhibited distinct juvenile facies, characterized by a lack of complex emotional expression, a fixed gaze, and an overall immature facial gestalt. These facial features were first observed in patients with MRD22. These variations may reflect the influence of modifying genes or environmental factors and highlight the crucial role of genetic testing in disease diagnosis.

The de novo *ZBTB18* variant NM_205768.3: c.1474delA (p.Arg492Aspfs∗11) identified in our patient has never been reported in previous studies. It is predicted to cause a premature termination codon that would trigger nonsense‐mediated mRNA decay, leading to impaired excitatory synaptic maturation and cognitive dysfunction in *ZBTB18* haploinsufficiency. This mechanism aligns with established models of synaptic and cognitive dysfunction in *ZBTB18* deficiency [26]. These findings revealed the genetic etiology of the neurodevelopmental disorder in our patient.

## 5. Conclusion

In summary, this study presents a female patient with a de novo *ZBTB18* frameshift variant (c.1474delA (p.Arg492Aspfs∗11)), who exhibited global developmental delay, epilepsy, unexplained ankle hypertonia, and unique juvenile facies. Notably, the patient′s seizures were sucessfully controlled with perampanel, which may provide a therapeutic insight for drug‐resistant epilepsy in MRD22. Our findings expand the genotypic and phenotypic spectrum of *ZBTB18*‐associated MRD22, providing novel insights into the clinical management of MRD22 and underscoring the critical role of genetic testing for diagnosis.

**Nomenclature**
MRD22: intellectual developmental disorder, autosomal dominant 22
ID: intellectual disability
WES: whole exome sequencing
MRI: magnetic resonance imaging
EEG: electroencephalogram

## Author Contributions

Yan Wu and Bei Li synthesized the clinical data; Yan Wu and Liang Liu analyzed the clinical and genetic data; Dongjing Li and Yan Wang drafted the manuscript.

## Funding

This study was supported by the Shaanxi Provincial Key Research and Development Program (Grant No. 2023‐ZDLSF‐19).

## Disclosure

All authors reviewed and approved the final manuscript.

## Ethics Statement

Written informed consent for publication was obtained from her parents. This study strictly adheres to the Helsinki Declaration and has been approved by the Hospital Ethics Committee.

## Conflicts of Interest

The authors declare no conflicts of interest.

## Data Availability Statement

The data that support the findings of this study are available from the corresponding authors upon reasonable request.

## Supporting information


**Supporting Information** Additional supporting information can be found online in the Supporting Information section. **Supporting Information.** Table S1: The treatment plan and timeline of the patient.
